# Movements of Medical Men

**Published:** 1841-06

**Authors:** 


### MOVEMENTS OF MEDICAL MEN.

The distinguished American Surgeon, Dr. Mott, after a residence of several years, in Paris, has returned to New York, his native place, and will, it is said, accept the chair of Surgery in the University of that city.

Dr. Oliver, of New Hampshire, has resigned, after one session, the professorship of Materia Medica in the Medical College of Ohio, and returned home.

We are happy to be able to announce the safe arrival in Europe, of our colleague Professor Caldwell. D.

